# The Three Cs of Integrated Tuberculosis-Diabetes Modules: A Competency-Based Medical Education Framework

**DOI:** 10.7759/cureus.115635

**Published:** 2026-09-02

**Authors:** Dinesh K Badyal, Sangeetha Mohan, Girish Joseph, Ashima Arora, Neena Bhatti, Prabhjot Kaur

**Affiliations:** 1 Pharmacology and Therapeutics, Christian Medical College & Hospital, Ludhiana, IND; 2 Microbiology, Christian Medical College & Hospital, Ludhiana, IND; 3 Medicine, Christian Medical College & Hospital, Ludhiana, IND; 4 Pathology, Christian Medical College & Hospital, Ludhiana, IND

**Keywords:** competency-based medical education, diabetes mellitus, integrated curriculum, medical education, tuberculosis

## Abstract

The introduction of Competency-Based Medical Education (CBME) in India emphasizes outcome-oriented training and provides a framework for implementing integrated teaching strategies. Traditional discipline-based medical education often limits the ability of learners to integrate knowledge across domains required to manage such complex comorbidities. Evidence suggests that integrated teaching approaches improve knowledge acquisition, clinical reasoning, and learner engagement.

The coexistence of tuberculosis (TB) and diabetes mellitus (DM) represents a significant and evolving public health challenge, particularly in high-burden countries such as India. Diabetes increases the risk of developing active TB and is associated with adverse treatment outcomes, including delayed sputum conversion, treatment failure, relapse, and increased mortality. This bidirectional interaction necessitates an integrated approach to both clinical management and medical education for TB and DM. This review synthesizes existing literature on the epidemiological and clinical interplay between TB and DM, along with educational frameworks supporting integration. This review of literature additionally presents a curriculum innovation comprising structured aligned integrated teaching (AIT) modules that integrate TB and DM across the MBBS curriculum using both horizontal and vertical integration strategies. The proposed framework may serve as a practical model for institutions implementing CBME. Adoption of structured integrated modules may enhance undergraduate medical training and better prepare future physicians for managing TB-DM comorbidity in high-burden settings.

## Introduction and background

The comorbidity of tuberculosis (TB) and diabetes mellitus (DM) represents a significant and evolving public health concern, particularly in high-burden countries such as India. India contributes nearly 27% of the global TB burden, a figure that has remained consistently high across successive global reporting cycles [1,2], while simultaneously harboring one of the largest populations of individuals living with diabetes, estimated to exceed 77 million, with projections indicating continued regional growth over the coming decades [3,4].

A growing body of evidence demonstrates that DM increases the risk of developing active TB by approximately two- to threefold, an association attributed in part to impaired innate and adaptive immune responses, including defective macrophage function, delayed T-cell-mediated immunity, and altered cytokine signaling in hyperglycemic states [5-8]. This relationship also appears to run in the opposite direction: TB itself has been associated with impaired glucose tolerance and new-onset hyperglycemia, likely mediated by stress-induced hormonal changes and the systemic inflammatory response to infection, underscoring the genuinely bidirectional nature of the comorbidity [9,10]. Beyond increased susceptibility, diabetes adversely influences the clinical course of TB, contributing to delayed sputum conversion, higher rates of treatment failure, relapse, and increased mortality [11-13]. Indian studies further highlight this association, reporting diabetes prevalence exceeding 20% among patients with TB in certain regions, with hospital- and cohort-based studies from across the country corroborating the high burden of TB-DM comorbidity and its adverse effect on treatment outcomes [14-16].

The close biological and clinical relationship between TB and DM highlights the need for integrated teaching of these conditions. Their management requires learners to connect concepts from microbiology, pathology, pharmacology, endocrinology, clinical medicine, and public health, including bidirectional screening, glycemic control, drug-related adverse effects, patient counseling, and long-term follow-up [5-16]. When these concepts are taught separately across different disciplines and phases, opportunities to develop a coherent understanding of TB-DM comorbidity may be missed. An integrated curriculum can therefore mirror real-world clinical practice by connecting these competencies across disciplines and progressively reinforcing them throughout the MBBS program [17,18].

## Review

Concept

Conventional medical education in India has historically followed a discipline-based model, wherein basic sciences and clinical subjects are taught in relative isolation. This compartmentalization often limits learners' ability to integrate knowledge across domains, particularly in conditions such as TB and DM that span microbiology, pharmacology, endocrinology, and public health. Integrated teaching approaches have been shown to address this gap by promoting contextual learning, reducing redundancy, and strengthening conceptual understanding across disciplines [17,18]. Educational studies demonstrate that integration-based models are associated with improved student engagement and significant gains in knowledge acquisition, as reflected in structured competency-based modules where post-module assessment scores and learner confidence improved significantly following implementation [17,19,20].

Curriculum integration extends beyond simply combining teaching sessions and instead represents a deliberate educational strategy that promotes meaningful connections between disciplines. Harden's framework for curriculum integration emphasizes progressively reducing disciplinary boundaries while preserving essential subject expertise, enabling learners to construct knowledge within authentic clinical contexts rather than as isolated facts [18]. Within Competency-Based Medical Education (CBME), integration is further strengthened through constructive alignment, whereby learning outcomes, instructional strategies, and assessment are intentionally aligned to facilitate competency acquisition [21]. Such an approach supports the development of clinical reasoning by encouraging learners to integrate anatomical, physiological, microbiological, pharmacological, pathological, and public health concepts while managing a single clinical condition. Consequently, integrated curricula are increasingly recognized as an effective means of improving knowledge retention, contextual understanding, and learner engagement in undergraduate medical education. A prospective educational interventional study involving 150 Phase I MBBS students evaluated an integrated teaching approach using a pre-test/post-test design and reported significant improvements in knowledge scores across Anatomy, Physiology, and Biochemistry, along with high student satisfaction [19].

Published literature describing structured curriculum models integrating TB and DM across the MBBS program using both horizontal and vertical integration within the CBME framework appears limited. Recognizing this bidirectional interaction between TB and DM, the World Health Organization has recommended collaborative frameworks for screening and joint management of TB and DM [1].

The introduction of CBME in India, most recently reaffirmed and updated through national curriculum guidelines, marked a major shift toward outcome-oriented training designed to produce an “Indian Medical Graduate” capable of addressing community health needs [22,23]. CBME emphasizes the alignment of learning objectives, teaching-learning methods, and assessment strategies, ensuring that students acquire not only knowledge but also the skills and professional attributes required for clinical practice [21]. Within this framework, aligned integrated teaching (AIT) serves as a key strategy to operationalize curricular integration by linking competencies across disciplines and phases of training [24,25].

Despite the clear clinical need for coordinated TB-DM care, structured disease-focused approaches to integrating TB and DM within undergraduate medical education remain limited. This gap is particularly relevant in high-burden settings such as India, where learners need to develop competencies that span multiple disciplines and phases of training. In response to this need, we developed institution-level AIT modules on TB and DM to illustrate the practical application of the proposed framework within the Indian CBME context. TB and DM represent an ideal model for disease-based curriculum integration because they require simultaneous understanding of infectious diseases, endocrinology, pharmacology, pathology, microbiology, and community medicine. Clinical management extends beyond diagnosis and treatment, encompassing bidirectional screening, glycemic optimization, adverse drug monitoring, patient counseling, nutritional management, and long-term follow-up. These interconnected competencies are traditionally taught across multiple departments and academic phases, often resulting in fragmented learning. An integrated curriculum enables students to appreciate the continuum of patient care while reinforcing the collaborative nature of multidisciplinary clinical practice. Such an approach mirrors contemporary recommendations for collaborative TB-DM management and prepares learners for managing increasingly complex multimorbid patients encountered in routine practice [1,11-16].

This article is a review of literature with a curriculum innovation component. It synthesizes the existing literature on the clinical and educational rationale for integrating TB and DM and describes an institution-developed AIT framework based on this evidence. The curriculum component illustrates the practical application of the proposed framework within the Indian CBME context and is not intended as a formal evaluation of educational effectiveness.

Conduct

The AIT module on TB was developed and implemented in accordance with the CBME curriculum prescribed by the National Medical Commission. The module was structured across Phase I, Phase II, and Phase III (Part I), ensuring both horizontal and vertical integration of competencies across disciplines.

The design process involved systematic identification of overlapping competencies, designation of lead departments for each phase, and selection of appropriate integration methods such as nesting and sharing, consistent with the module-development framework described in comparable AIT modules [24,25]. The phase-wise structure of the module is summarized in Tables 1, 2.

**Table 1:** Integrated module of tuberculosis, Phase 1 and Phase 2 MBBS Nesting: a competency from one subject is taught within a session led by another subject without added session time. Sharing: subjects jointly contribute session time to teach overlapping competencies. Abbreviations: LGT: Large Group Teaching; SGT: Small Group Teaching; AIT: Aligned Integrated Teaching; AN: Anatomy; PY: Physiology; CT: Chest Medicine; BI: Biochemistry; PE: Pediatrics; MI: Microbiology; CM: Community Medicine; PA: Pathology; PH: Pharmacology; TB: Tuberculosis. Source: Competency codes are based on the Competency Based Undergraduate Curriculum for the Indian Medical Graduate, Medical Council of India, 2018 [26].

Phase	TL method	Lead subject	Competency number	Method of integration	Session time (hours)
Phase 1	LGT	Anatomy	AN21.9, PY6.1	Nesting	1
Phase 1	SGT	Anatomy	AN23.1-23.7, PY6.2	Nesting	2
Phase 1	LGT	Anatomy	AN24.1, PY6.3	Nesting	1
Phase 1	SGT	Anatomy	AN24.2-24.6, PY6.3	Nesting	2
Phase 1	SGT	Anatomy	AN24.2-24.6, PY6.2	Nesting	2
Phase 1	SGT	Anatomy	AN24.1, PY6.2	Nesting	1
Phase 1	LGT	Anatomy	AN24.3, PY6.8	Nesting	1
Phase 1	SGT	Anatomy	AN24.2-24.6, PY6.1	Nesting	2
Phase 1	LGT	Physiology	PY6.7, CT2.11	Sharing	2
Phase 1	SGT	Biochemistry	BI11.5, PE34.10	Sharing	2
Phase 2	SGT	Microbiology	MI6.3, MI6.1, CT1.2	Sharing	2
Phase 2	LGT	Microbiology	TB recap	Nesting	1
Phase 2	LGT	Community medicine	CM8.1, CT1.1	Sharing	1
Phase 2	LGT	Pathology	PA4.3, PA19.2, MI6.3	Sharing	1
Phase 2	LGT	Pathology	PA24.5, PA26.4, MI5.1	Nesting	1
Phase 2	SGT	Pathology	PA26.4, MI1.2, CT1.7, PE34.2	Sharing	2
Phase 2	LGT	Pharmacology	PH1.44, PH1.45	Sharing	2
Phase 2	LGT	Microbiology	MI1.69, PE34.6, PE34.7, CM10.5	Sharing	1
Phase 2	LGT	Community medicine	CM8.3, PE34.3, PH1.55	Sharing	1
Phase 2	LGT	Pathology	PA33.1, PA35.1, MI5.1	Nesting	1
Phase 2	SGT	Pathology	PA19.3, MI1.3	Sharing	2
Phase 2	SGT	Microbiology	MI6.1, MI6.2, PA26.4, CT1.7, PE34.2	Sharing	2
Phase 2	SGT	Pathology	AIT TB (long case) assessment	Assessment	2

**Table 2:** Integrated module of tuberculosis: Phase 3 Part 1 and Part 2 MBBS Nesting: a competency from one subject is taught within a session led by another subject without added session time. Sharing: subjects jointly contribute session time to teach overlapping competencies. Abbreviations: LGT: Large Group Teaching; SGT: Small Group Teaching; AIT: Aligned Integrated Teaching; CT: Chest Medicine; PE: Pediatrics; MI: Microbiology; CM: Community Medicine; TB: Tuberculosis; OG: Obstetrics & Gynecology; OR: Orthopedics; PA: Pathology; PH: Pharmacology. Source: Competency codes are based on the Competency Based Undergraduate Curriculum for the Indian Medical Graduate, Medical Council of India, 2018 [26].

Phase	TL method	Lead subject	Competency number	Method of integration	Session time (hours)
Phase 3 Part 1	LGT	Community medicine	TB recap	Nesting	1
Phase 3 Part 1	LGT	Community medicine	CM8.1, CT1.1, CT1.2	Sharing	1
Phase 3 Part 1	SGT	Obstetrics & Gynaecology	OG27.2, MI6.1	Sharing	2
Phase 3 Part 1	LGT	Pediatrics	PE34.1, PE34.2, MI6.1, MI6.2	Sharing	1
Phase 3 Part 1	SGT	Orthopedics	OR3.1, PA33.1	Nesting	2
Phase 3 Part 1	SGT	Orthopedics	OR4.1, MI6.1	Sharing	2
Phase 3 Part 1	SGT	Medicine	CT1.3, CT1.4, CT1.14-1.15, PH1.44, PH1.45	Sharing	2
Phase 3 Part 1	LGT	Community medicine	CM8.3, CM8.5, PE34.3	Sharing	1
Phase 3 Part 1	LGT	Medicine	CT1.17, CT1.13-1.16, CM10.5	Nesting	1
Phase 3 Part 1	Assessment	Ophthalmology	Test - AIT TB	Assessment	2
Phase 3 Part 2	LGT	Medicine	TB recap	Nesting	1
Phase 3 Part 2	LGT	Respiratory medicine	CT1.8, CT1.9	Sharing	1
Phase 3 Part 2	SGT	Respiratory medicine	CT1.10, CT1.11	Sharing	2
Phase 3 Part 2	LGT	Pediatrics	PE34.1, PE34.2, MI6.1	Sharing	1
Phase 3 Part 2	SGT	Orthopedics (recap)	OR3.1, PA33.1	Nesting	2
Phase 3 Part 2	LGT	Medicine	Reflections	Reflection	2

Integration was operationalized using three complementary approaches - nesting, sharing, and correlation - to facilitate coordinated learning across disciplines. Nesting involved embedding relevant components across disciplines, with all participating disciplines made aware of the relevant competencies. Sharing involved joint teaching sessions addressing common learning objectives, thereby reducing redundancy and reinforcing key concepts across disciplines. Correlation was achieved through linker cases that connected concepts across disciplines using authentic clinical scenarios. These linker-case discussions anchored learning in clinical contexts and enabled students to synthesize knowledge from multiple domains. This phased structure supported progressive learning, allowing students to build foundational knowledge and subsequently apply it to clinical contexts. An example illustrating the methods of integration used is presented in Figure 1.

**Figure 1:**
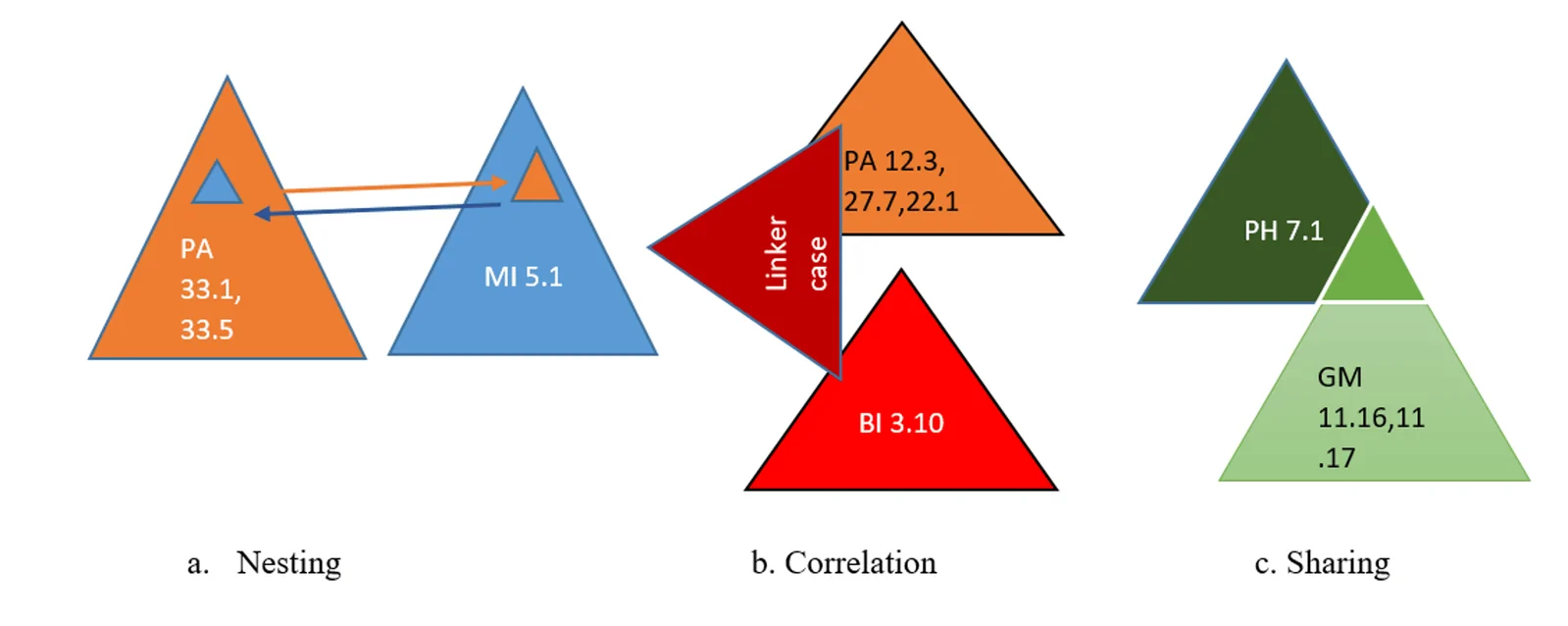
Integration strategies used in the Aligned Integrated Teaching module Schematic representation of the three integration strategies: (a) Nesting, (b) Correlation using a linker case, and (c) Sharing between departments. Numbers within the triangles indicate the corresponding integrated teaching sessions. Abbreviations: PA: Pathology; PH: Pharmacology; MI: Microbiology; BI: Biochemistry; GM: General Medicine. Image credits: Neena Bhatti using Microsoft Word (Microsoft Corporation, Redmond, Washington).

The design philosophy also incorporates principles of progressive competency development. Foundational concepts introduced during the preclinical years are intentionally revisited during clinical training with increasing complexity, allowing learners to progressively integrate biomedical knowledge with clinical reasoning and patient management. This longitudinal reinforcement is consistent with competency-based educational principles that emphasize repeated exposure, contextual learning, and progressive entrustment of professional responsibilities [21,27,28]. Rather than functioning as isolated teaching sessions, each integrated module contributes to an interconnected learning pathway extending across the undergraduate curriculum.

The AIT module on DM was similarly developed in alignment with CBME guidelines [26]. The module spans Phase I, Phase II, and Phase III (Parts 1 and 2), ensuring continuity of learning from foundational sciences to advanced clinical management. The design emphasized identification of shared competencies across disciplines, followed by allocation of lead departments and selection of integration strategies, including correlation, nesting, sharing, and case-based learning. This structured approach ensured alignment between learning objectives, instructional methods, and assessment strategies. The phase-wise organization of the module is presented in Tables 3, 4.

**Table 3:** Integrated module of diabetes mellitus: Phase 1 and Phase 2 MBBS Nesting: a competency from one subject is taught within a session led by another subject without added session time. Sharing: subjects jointly contribute session time to teach overlapping competencies. Correlation: related competencies from different subjects are taught in sequence within the same session. Linker: a brief session explicitly connecting a previously taught competency to a new one. LGT: Large Group Teaching; SGT: Small Group Teaching; AIT: Aligned Integrated Teaching; AN: Anatomy; PY: Physiology; BI: Biochemistry; PE: Pediatrics; IM: Internal Medicine; CM: Community Medicine; PA: Pathology; PH: Pharmacology. Source: Competency codes are based on the Competency Based Undergraduate Curriculum for the Indian Medical Graduate, Medical Council of India, 2018 [26].

Phase	TL method	Lead subject	Competency number	Method of integration	Session time (hours)
Phase 1	LGT + SGT	Anatomy	AN47.5.1-2, PY8.2.1-5	Correlation	3
Phase 1	Lecture	Anatomy	AN52.1.1-2, PY8.2.50-56	Nesting	1
Phase 1	LGT + SGT	Physiology	PY8.2.1-5, PY8.2.50-57, BI3.8-9	Nesting	2
Phase 1	SGT	Community medicine	CM8.2, BI8.3, BI11.23, IM11.7	Sharing	2
Phase 1	LGT + SGT	Biochemistry	BI13.8-10, BI16.1, PA32.4	Correlation	3
Phase 1	LGT + SGT	Physiology	PY8.2.57-58, IM11.7, IM11.22	Nesting	2
Phase 1	Lecture	Anatomy	AN52.6.1-2, PA32.4	Nesting	1
Phase 1	Lecture	Physiology	PY8.4.23-32, AN52.1.1, BI3.8	Nesting	1
Phase 1	LGT + SGT	Biochemistry	BI7.7, BI11.17, PY8.4, IM11.11-13	Sharing	2
Phase 1	SGT	Physiology	PY8.4.30-32, BI3.8	Linker	1
Phase 2	Lecture	Physiology	Recap	Nesting	1
Phase 2	Lecture	Pathology	PA32.4.1, PA32.4.3-5, IM11.1.1-3, PE33.4	Nesting	1
Phase 2	Lecture	Pathology	PA32.4.6-8, IM11.5	Nesting	1
Phase 2	LGT	Community medicine	CM8.2, PA32.4.2, IM11.2-3	Nesting	2
Phase 2	LGT + SGT	Pharmacology	PH1.36.1-4, PY8.2.52-55, IM11.19	Sharing	2

**Table 4:** Integrated module of diabetes mellitus: Phase 3 Part 1 and Part 2 MBBS Nesting: a competency from one subject is taught within a session led by another subject without added session time. Sharing: subjects jointly contribute session time to teach overlapping competencies. Correlation: related competencies from different subjects are taught in sequence within the same session. Linker: a brief session explicitly connecting a previously taught competency to a new one. LGT: Large Group Teaching; SGT: Small Group Teaching; AIT: Aligned Integrated Teaching; PE: Pediatrics; IM: Internal Medicine; DR: Dermatology & Venereology; OP: Ophthalmology; SU: Surgery; CM: Community Medicine; PH: Pharmacology. Source: Competency codes are based on the Competency Based Undergraduate Curriculum for the Indian Medical Graduate, Medical Council of India, 2018 [26].

Phase	TL method	Lead subject	Competency number	Method of integration	Session time (hours)
Phase 3 Part 1	Lecture	Community medicine	Recap	Nesting	1
Phase 3 Part 1	SGT	Internal medicine	IM11.1-3, PE33.4, DR18.1	Correlation	2
Phase 3 Part 1	LGT	Internal medicine	IM11.4, CM8.2	Sharing	1
Phase 3 Part 1	SDL	Internal medicine	IM11.5-6, OP8.2, PE33.4	Nesting	3
Phase 3 Part 1	Bedside teaching	Internal medicine	IM11.7, PH1.36.8	Nesting	2
Phase 3 Part 1	Bedside teaching	Internal medicine	IM11.8, SU27.2, OP7.3, OP8.3	Sharing	2
Phase 3 Part 1	SGT	Internal medicine	IM11.9, IM11.10	Sharing	2
Phase 3 Part 1	SGT	Internal medicine	IM11.11, PE33.5, SU9.1	Correlation	2
Phase 3 Part 1	Bedside teaching	Pediatrics	IM11.12-13, PE33.5	Sharing	2
Phase 3 Part 2	LGT	Pediatrics	IM11.14, PE33.4, DR18.1	Nesting	1
Phase 3 Part 2	Lecture	Internal medicine	IM11.15, PE33.4, PH1.36	Correlation	1
Phase 3 Part 2	SGT	Internal medicine	IM11.16, PH1.38	Nesting	2
Phase 3 Part 2	SGT	Internal medicine	IM11.17, CM8.2, PH1.36, OP8.2-3	Nesting	2
Phase 3 Part 2	LGT	Pediatrics	IM11.18, PE33.4-33.5	Sharing	1
Phase 3 Part 2	Lecture	Internal medicine	IM11.19, PE33.5	Sharing	1
Phase 3 Part 2	LGT	Internal medicine	IM11.20, PE33.5	Nesting	1
Phase 3 Part 2	SGT	Internal medicine	IM11.21, PH1.36	Correlation	2
Phase 3 Part 2	SGT	Obstetrics & Gynaecology	IM11.22, OBG12.3	Nesting	2
Phase 3 Part 2	LGT	Internal medicine	IM11.23, IM11.13, PE33.5	Nesting	1
Phase 3 Part 2	Lecture	Pediatrics	IM11.24, PE33.4	Sharing	1

The diabetes module is structured to reflect the systemic nature of the disease, integrating basic science concepts with clinical and public health dimensions across all phases of training. Early phases focus on foundational knowledge such as pancreatic structure, insulin physiology, and metabolic pathways, while later phases address clinical management, complications, and interdisciplinary care. The objective of this article is to describe the conceptualization, competency mapping, and implementation framework of these modules rather than to evaluate their educational effectiveness. Formal evaluation of learner outcomes and faculty perceptions will be reported separately. The conceptual relationship between the three components of the proposed framework - Concept, Conduct, and Curriculum Placement - and their contribution to competency development is illustrated in Figure 2.

**Figure 2:**
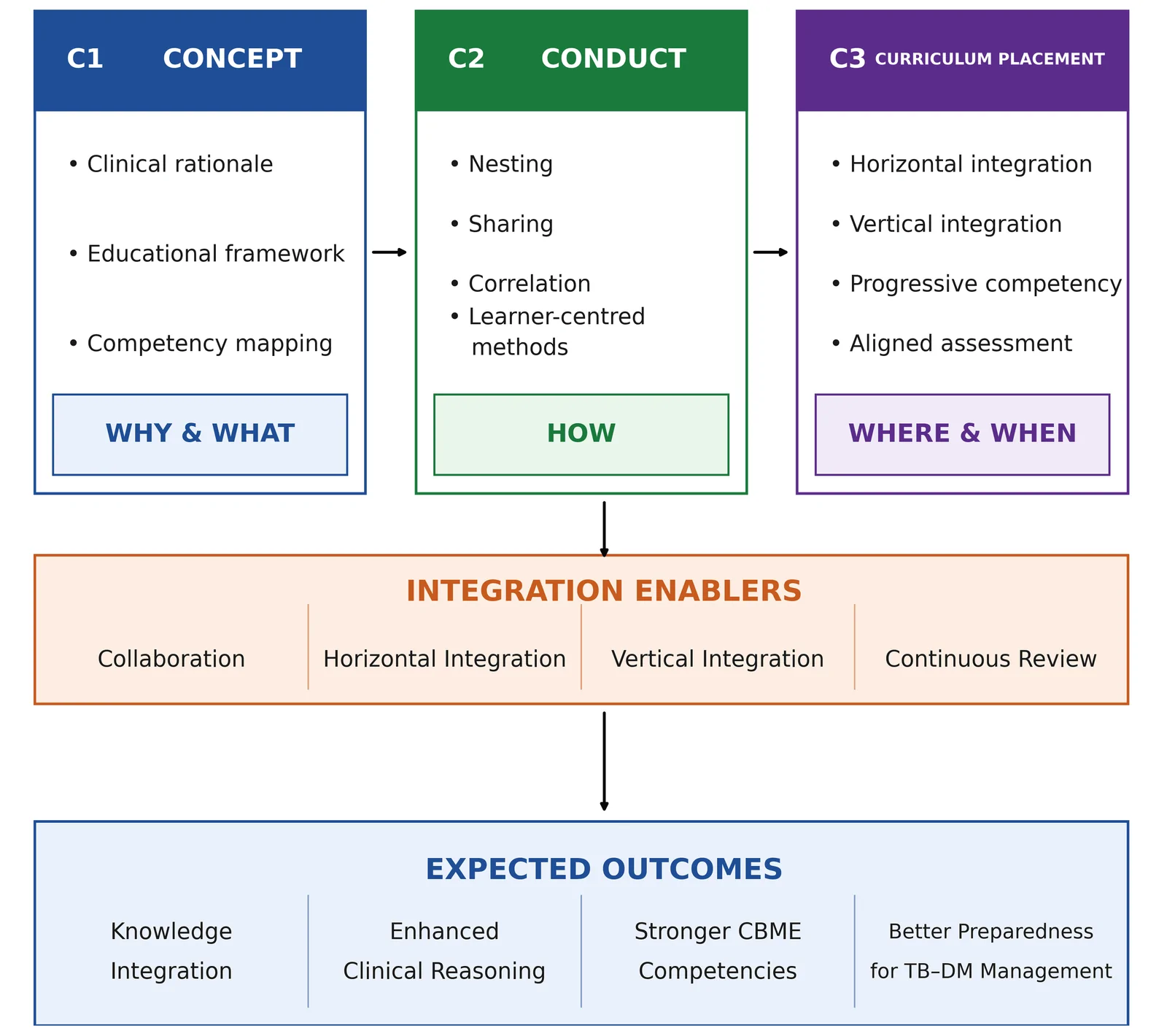
Conceptual framework of the Three Cs of integrated TB–DM modules Conceptual framework illustrating the purpose, design (Concept–Conduct–Curriculum Placement), integration enablers, and expected educational outcomes of the integrated TB–DM module aligned with competency-based medical education (CBME). Image Credit: Girish Joseph using Microsoft PowerPoint (Microsoft Corporation, Redmond, Washington) and Canva (Canva Pty Ltd., Sydney, Australia).

Curriculum placement and integration

The various planned sessions are added to the annual schedule as shown in Figure 3.

**Figure 3:**
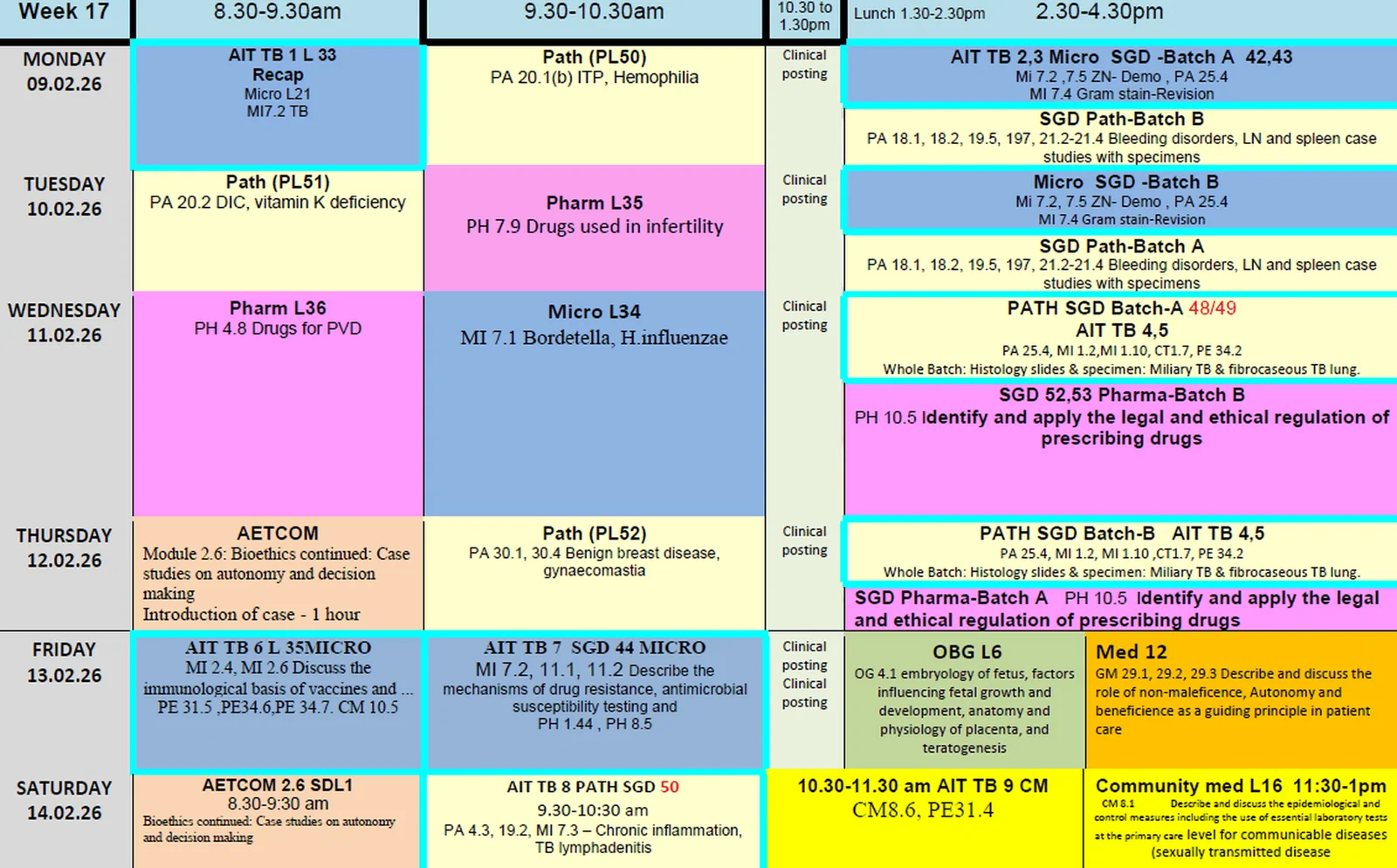
Curriculum placement of aligned integrated teaching sessions in the MBBS timetable Weekly timetable illustrating the placement of aligned integrated teaching (AIT) sessions on tuberculosis alongside routine departmental teaching and clinical postings. Image Credit: Dinesh K. Badyal using Microsoft Word (Microsoft Corporation, Redmond, Washington).

The color coding is being used to highlight AIT sessions versus other teaching sessions. Along with this, departments are given entire AIT modules to plan their sessions. Lead departments plan and execute the sessions. Integration strategies were applied in a complementary manner. This structured, phase-wise integration supports progressive learning and reflects real-world clinical practice, where effective management of diabetes requires coordinated understanding of physiology, pharmacology, pathology, and clinical medicine.

Integration is implemented through horizontal and vertical approaches. Horizontal integration aligns related subjects within the same phase, facilitating simultaneous learning of interconnected concepts. Vertical integration links basic sciences with clinical disciplines across phases, reinforcing foundational knowledge through clinical application [18,19,27]. While implementation requires faculty training, coordination, and institutional support, integrated approaches have been shown to improve both learning outcomes and interdisciplinary collaboration [27-30].

Successful implementation of integrated curricula requires sustained collaboration among departments throughout planning, delivery, assessment, and continuous quality improvement. Faculty members must collectively identify overlapping competencies, define shared learning outcomes, coordinate teaching schedules, and develop assessment strategies that evaluate integrated clinical performance rather than discipline-specific factual recall. Such collaborative curriculum planning also promotes faculty development by fostering interdisciplinary dialogue and encouraging educators to adopt learner-centered instructional approaches consistent with CBME principles [27-30]. Institutional commitment therefore remains fundamental for the sustainability of integrated educational initiatives. However, implementation presents several challenges. Faculty trained in traditional systems may require structured development programs to effectively design and deliver integrated sessions, a process that also intersects with faculty members' own professional identity as educators alongside their clinical and research roles [27-30]. Institutional coordination across departments, including scheduling and collaborative planning, can also be complex and requires administrative support [27-29]. Additionally, assessment methods must evolve to evaluate integrated competencies rather than isolated subject knowledge, following evaluation approaches such as observed structured assessments and structured learner feedback used in comparable competency-based modules [19,20,27]. Although designed within the Indian CBME framework, the principles of competency mapping, phased integration, and interdisciplinary teaching described in this article are adaptable to other competency-based undergraduate medical curricula. Despite these advantages, implementation should be regarded as an iterative process requiring regular curriculum review and stakeholder feedback. Continuous evaluation enables institutions to identify redundancy, curricular gaps, scheduling conflicts, and assessment challenges while ensuring alignment with evolving competency frameworks. Faculty development workshops, structured learner feedback, and periodic curriculum mapping can facilitate continuous refinement of integrated modules and support their long-term sustainability [27-30].

Limitations

As a review of literature, this article does not employ a systematic literature search methodology and therefore may not include all available evidence regarding integrated medical education. Furthermore, the educational modules presented represent the experience of a single institution within the Indian CBME framework. The curriculum component should therefore be regarded as an institution-level implementation framework rather than evidence of educational effectiveness. Formal evaluation of learner and faculty outcomes and multicenter validation will be required to establish its effectiveness and generalizability. Although the proposed framework is potentially adaptable to other educational settings, formal multicenter evaluation will be necessary to determine its effectiveness and generalizability.

Future directions

Future research should focus on evaluating the educational effectiveness of the proposed integrated TB-DM modules through prospective multicenter studies involving diverse medical institutions. Outcomes should include knowledge acquisition, clinical reasoning, competency attainment, learner satisfaction, faculty perceptions, and long-term retention of integrated competencies. Comparative studies assessing integrated curricula against conventional discipline-based teaching would further strengthen the evidence base. Future work should also explore the incorporation of simulation-based learning, workplace-based assessment, digital learning platforms, and interprofessional education to enhance learner engagement and authentic competency assessment. Finally, adaptation of the proposed Three Cs framework (Concept, Conduct, and Curriculum Placement) to other multimorbid and interdisciplinary conditions could facilitate broader implementation of competency-based integrated curricula across undergraduate medical education.

## Conclusions

Existing literature supports integrated teaching as a means of improving academic performance, conceptual understanding, learner satisfaction, and application of knowledge in clinical settings. Within CBME, AIT aligns competencies, teaching methods, and assessment while integrating public health perspectives relevant to TB-DM. This approach promotes interdisciplinary learning and prepares students to manage complex comorbid conditions.

AIT provides a practical framework for competency-based integrated education, although sustained implementation requires faculty development, institutional support, and ongoing curriculum refinement. The Three Cs framework may also be adapted to other chronic and communicable diseases requiring multidisciplinary management, providing a broader model for strengthening integrated undergraduate medical education.
